# Repurposing Antifungals for Host-Directed Antiviral Therapy?

**DOI:** 10.3390/ph15020212

**Published:** 2022-02-10

**Authors:** Sebastian Schloer, Jonas Goretzko, Ursula Rescher

**Affiliations:** 1 Institute-Associated Research Group “Regulatory Mechanisms of Inflammation”, Institute of Medical Biochemistry, Center for Molecular Biology of Inflammation, and “Cells in Motion” Interfaculty Centre, University of Muenster, Von-Esmarch-Str. 56, D-48149 Muenster, Germany; j_gore01@uni-muenster.de; 2 Leibniz Institute for Experimental Virology, D-20251 Hamburg, Germany

**Keywords:** antifungals, host-directed drug therapy, drug repurposing, azoles, polyenes, echinocandins, viral infections

## Abstract

Because of their epidemic and pandemic potential, emerging viruses are a major threat to global healthcare systems. While vaccination is in general a straightforward approach to prevent viral infections, immunization can also cause escape mutants that hide from immune cell and antibody detection. Thus, other approaches than immunization are critical for the management and control of viral infections. Viruses are prone to mutations leading to the rapid emergence of resistant strains upon treatment with direct antivirals. In contrast to the direct interference with pathogen components, host-directed therapies aim to target host factors that are essential for the pathogenic replication cycle or to improve the host defense mechanisms, thus circumventing resistance. These relatively new approaches are often based on the repurposing of drugs which are already licensed for the treatment of other unrelated diseases. Here, we summarize what is known about the mechanisms and modes of action for a potential use of antifungals as repurposed host-directed anti-infectives for the therapeutic intervention to control viral infections.

## 1. Introduction

Throughout history, the world’s population has been impacted by virus outbreaks, epidemics and pandemics. The recently emerged severe acute respiratory syndrome coronavirus 2 (SARS-CoV-2) and the resulting coronavirus disease 2019 (COVID-19) have impressively shown how quickly the spread of a novel virus becomes a major threat for public health worldwide [1]. The ongoing pandemic with high mortality and morbidity rates highlights the urgent need for safe and fast development of new pharmaceutics to combat newly emerging viral infections [2,3]. While the ultrafast development of vaccines to fight SARS-CoV-2 has been an unprecedented success story, the high mutation rates leading to rapidly changing viral genomes also require a continuous update of vaccines [4,5,6]. Moreover, antigenic shift, i.e., the recombination of viral genomes and the appearance of new viral subtypes, is a great public health concern [5,6]. Thus, antiviral drugs are essential for the management and control of viral infections to fill the gap until virus-specific routine immunization strategies are available.

**Figure 1:**
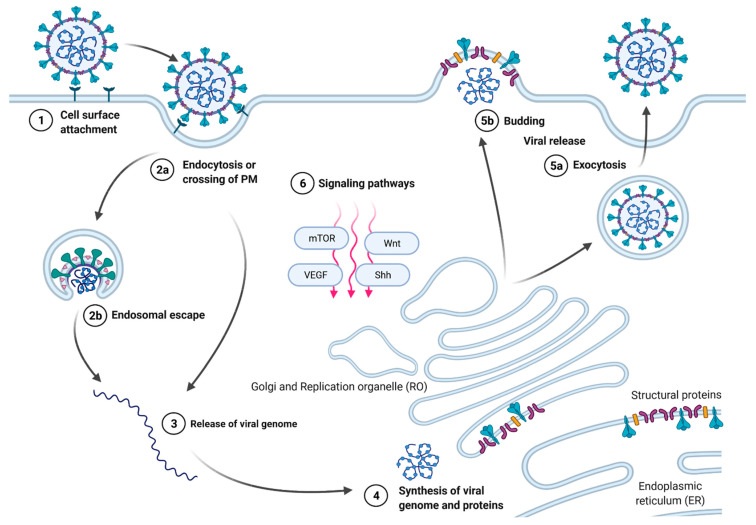
**Basic steps of the replication cycle of viruses in mammalian cells.** Upon attachment of the virus to receptors on the host cell surface (1), viruses cross cellular membranes to gain access to the host cell, either by penetration (for nonenveloped viruses) of or fusion (for enveloped viruses) with the plasma membrane (2a) or endosomes (2b), and the viral genome is released into the host cell (3). Viral replication depends on the synthesis of viral components using the existing or modified host cell organelles (4) and the release of the newly assembled virions from the host cell via either exocytosis of virion-containing vesicles (5a) or budding (5b). To support the viral replication, viruses modulate cellular signaling pathways, such as the Wnt, Shh, VEGF, and mTORC1 signal transduction pathways (6). Repurposed drugs might target (i) viral interaction with cellular membranes (during virus internalization, assembly, release), (ii) translation and modification of viral proteins, and (iii) cellular signaling pathways hijacked by the virus to suppress virus detection and destruction and promote viral assembly and release. Adapted from “Coronavirus Replication Cycle”, by BioRender.com (2020). Retrieved from https://app.biorender.com/biorender-templates (accessed date on 15 December 2021).

While drugs that directly act on viral components (direct antivirals) usually offer safe and effective treatment options, they also come with the risk of emerging resistances, as seen by the tremendously fast and effective adaptation of influenza A virus (IAV) to the viral neuraminidase inhibitor oseltamivir [7,8]. Although viruses come in all shapes and sizes and have confusingly diverse replication cycles, the common denominator is the absolute dependence on a host cell for their propagation. As a consequence, all viruses exploit fundamental cellular processes to gain access to the cellular replication and transport machinery for the biosynthesis of the viral genome, virus proteins, and the assembly and release of virions. The common basic steps in infection cycles of viruses are shown in Figure 1. Notably, virus replication depends on (i) the interaction with cellular membranes (during virus internalization, assembly, release), (ii) translation and modification of viral proteins, and (iii) manipulation of cellular signaling pathways to suppress virus detection and destruction and promote viral assembly and release. Drugs that target these cellular functions will most likely also affect the propagation of a whole range of otherwise unrelated viruses. Thus, such host-directed therapeutic strategies could be advantageous as new antiviral approaches, especially for the threat posed by new emerging viruses [9]. Repurposing clinically approved drugs that have been developed for other indications but also target such cellular processes provides a particularly attractive strategy to move such treatments into the clinic faster and safer, as these drugs are already in use. Indeed, this repurposing strategy is currently being increasingly pursued in the fight against COVID-19 [10]. Here, we present an overview of the commonly used antifungals and review what is known about their antiviral potential and the putative molecular targets and mechanisms/modes of action in mammalian cells.

## 2. Antifungal Drugs

The synthetic small molecule antifungals are classified according to their targets and mechanisms of action, and comprise four major classes, the polyenes, pyrimidine analogs (flucytosine), azoles, and echinocandins (Table 1).

### 2.1. Polyenes—Disruptors of Fungal Membrane

Polyenes exert their antifungal activity by binding ergosterol in the fungal cell membrane. The resulting membrane disintegration by pore formation increases the permeability, and the membrane leakage leads to the subsequent death of the fungal cell. A well-known, highly effective polyene is amphotericin B, which is widely used in the clinics against invasive fungal infections. Amphotericin B was shown to also induce oxidative stress to fungal cells and modulate the immune system [33].

A potential antiviral capacity of amphotericin B has been reported against a variety of viruses, including human immunodeficiency virus (HIV) [14], Japanese encephalitis virus (JEV) [12], herpes simplex virus (HSV) [13], rubella virus [12] and vesicular stomatitis virus (VSV) [15]. Amphotericin B inhibits the infectivity of JEV in a concentration-dependent manner up to 200-fold at a postinfection step likely at viral replication and/or synthesis of viral proteins without affecting virus adsorption to host cell surfaces [12].

In vitro results highlight the possibility of using amphotericin B against HIV infections, providing a dual effect, against the virus itself and the opportunistic fungal infections that often accompany HIV infections due to the patients’ compromised immune system [14]. Furthermore, amphotericin B potentiated the antiviral efficacy of acyclovir against pseudorabies virus (PRV) without a direct effect on PRV replication in the absence of acyclovir [34]. The mechanism of action remains unclear; however, a recent high-throughput virtual screening approach showed amphotericin B, among other antiparasitic drugs, to possess potential inhibitory features against 10 SARS-CoV-2 molecular targets including the RNA-dependent RNA polymerase [35].

### 2.2. Flucytosine—A Selective Inhibitor of Fungal Nuclear Acid Synthesis

Flucytosine as such has no antifungal capacity but is converted by the fungal enzyme cytosine deaminase (which is not present in mammalian cells) into 5-fluorouracil, which is further metabolized. The incorporation of 5-fluorouracil and its metabolites into fungal DNA and RNA then causes aberrant fungal RNA and DNA synthesis [36,37]. To our knowledge, there are no studies reporting antiviral properties of flucytosine yet.

### 2.3. Echinocandins—Noncompetitive Inhibitors of (1,3)-β-D-Glucan Synthase

Echinocandins attack the fungal cell wall by inhibiting the (1,3)-β-D-glucan synthesis and thereby triggering osmotic stress and subsequent cell lysis. They are fungicidal against molds and yeast (most *Candida* species) and are generally considered well-tolerable due to little adverse effects and drug–drug interactions. Therefore, they are the preferred treatment option for invasive candidiasis [38,39]. Micafungin is an FDA-approved echinocandin that exhibits broad antifungal activity against a variety of *Candida* species. Of note, a potential antiviral capacity against chikungunya virus (CHIKV) [18], enteroviruses [19] and dengue virus [20], among others, has been reported in recent years. Mosquito-borne CHIKV belongs to the alphaviruses and is a global health problem. Micafungin was able to attenuate the cytopathic effects of CHIKV, reduce viral replication, release and spread, and impair viral stability. Micafungin also had antiviral effects against the alphaviruses Sindbis virus (SINV) and Semliki Forest virus (SFV) [18]. Enterovirus 71 (EV71), the major causative agent of hand-foot-and-mouth disease (HFMD), was shown sensitive to micafungin treatment as well. Micafungin effectively diminished EV71 proliferation and replication already at a micromolar dose [19]. Against other enteroviruses like Coxsackievirus group B type 3 (CVB3) and human rhinovirus (HRV), its antiviral capacity was only moderate. The authors proposed a virion-independent mechanism of action targeting intracellular processes such as translation, polyprotein processing or replication [19]. Recently, micafungin and its analogs, caspofungin and anidulafungin, were suggested for treatment of dengue virus (DENV) infection. In this case, the mechanism of action depends on the direct binding to the envelope protein DENV-2, thereby destabilizing and destroying the virion [20]. Of note, recent in silico studies argued for a binding of the echinocandins micafungin and pneumocandin B0 to the 3C-like protease (3CLpro) of porcine epidemic diarrhea virus (PEDV), as well as to the severe acute respiratory syndrome coronavirus 2 (SARS-CoV-2) main protease (Mpro), with micafungin having a higher calculated binding affinity towards 3CLpro and pneumocandin B0 binding preferably to Mpro, warranting further investigation into the use of echinocandin-type antifungal drugs as antiviral agents that act via binding to defined viral molecular targets [21].

### 2.4. Azoles—Inhibitors of Ergosterol Biosynthesis

The antifungal azoles are classified by the numbers of nitrogen atoms in the azole ring and include imidazoles (e.g., ketoconazole, miconazole, and clotrimazole) and triazoles (e.g., itraconazole, posaconazole, and fluconazole) [40]. Their efficacy and relative safety have led to their widespread clinical use for the antifungal therapy against aspergillosis, candidiasis, and cryptococcosis [41]. While the clinical use of imidazoles (the only exception is ketoconazole) is limited to the treatment of superficial mycoses, triazoles are used for superficial and systemic fungal infections [42]. Azoles exert their antifungal activities through multiple modes of action. By inhibiting the two cytochrome P450 enzymes that function in the ergosterol biosynthesis (CYP51, lanosterol 14α-demethylase) and the conversion of ergostatrienol into ergostatetraenol, (CYP61, 22-desaturase), azoles lead to a depletion of ergosterol. The resulting accumulation of toxic sterol precursors impairs membrane fluidity, asymmetry, and membrane integrity in fungal cells [43].

Interestingly, some of the azoles (itraconazole, posaconazole, voriconazole, and ketoconazole) also impact the mammalian cholesterol metabolism at higher concentrations [44]. Itraconazole and posaconazole influence cellular cholesterol levels by impairing different pivotal steps in the cholesterol homeostasis. Both antifungals include (i) the inhibition of the lanosterol 14α-demethylase and thereby the homeostasis and de novo synthesis of cholesterol [45,46], (ii) the inhibition of the cholesterol-transferring membrane protein Niemann–Pick C1 (NPC1) that results in the accumulation of cholesterol in the endolysosomal system [47], and they further (iii) interact with the oxysterol-binding protein (OSBP), which blocks the shuttling of cholesterol and phosphatidylinositol-4-phosphate (PI4P) between membranes [31]. In the following we will exemplarily discuss the interaction of itraconazole with cellular proteins.

#### 2.4.1. Itraconazole Directly Interacts with the Endolysosomal Cholesterol Transporter NPC1

Niemann–Pick disease, type C1 (NPC1) protein NPC1 was first identified and characterized as a membrane protein that when mutated causes Niemann–Pick disease, type C1, a rare autosomal neurovisceral lipid storage disorder [48]. NPC1 protein is an endolysosomal integral membrane protein and mediates endolysosomal cholesterol transport [49]. A dysfunctional NPC1 protein, which is found mutated in 95% of the NPC patients, disturbs the intracellular lipid transport, leading to the excessive accumulation of lipid products including cholesterol in the endolysosomal compartment [48]. Interestingly, a possible function of NPC1 as a drug target in antiviral strategies has been explored in several recent publications [50,51]. The Ebola virus entry has been shown to directly rely on NPC1 function via binding of the Ebola virus glycoprotein (GP) to NPC1 [52]. The Ebola virus GP is cleaved by endosomal proteases to unmask the NPC1 binding site, and GP–NPC1 engagement within lysosomes promotes the viral escape into the host cytoplasm [53]. Consistent with this vital dependence of the Ebola virus replication on NPC1 protein, cells lacking NPC1 are nonpermissive for the virus entry and NPC1 knockout mice are protected from lethal Ebola virus infection [52]. Blocking NPC1 has also been reported to cause accumulation of the HIV-1 viral gag protein in the endolysosomal compartment [54,55], resulting in a profound suppression of virion release [55]. The finding that the HIV-1 accessory protein Nef induces host cell genes involved in cholesterol biosynthesis and homeostasis [56] emphasizes the strong dependency of HIV-1 on the host cell cholesterol levels, suggesting that NPC1 is a candidate drug target in the treatment of HIV-1 infections. Interestingly, NPC1 also emerged as a candidate drug target for other enveloped viruses, namely IAV and SARS-CoV-2. Viral replication rates were decreased in cells in which NPC1 was functionally blocked, and the increased endolysosomal cholesterol levels were suggested to interfere with the proper insertion of the fusogenic IAV hemagglutinin domains and the SARS-CoV-2 spike protein, thus affecting virus uncoating [23,24,25].

In many of these and other in vitro studies exploring the importance of NPC1 in diverse cellular functions, the cell-permeable hydrophobic polyamine U18666A, a small-molecule NPC1 inhibitor, was used [57,58]. However, the substantial toxicity of this compound limits a clinical use [59,60]. Of note, itraconazole has been shown to also directly bind and inhibit NPC1 [47], and thus might serve as an attractive candidate for NPC1 targeting strategies via drug repurposing. In favor of this notion, itraconazole-treated cells generated lesser IAV and Ebola virus progeny [25,28], and a beneficial treatment outcome was indeed confirmed in a mouse IAV infection model in vivo [25]. Itraconazole-mediated induction of type I interferons (IFNs), which is considered a fundamental step in establishing antiviral immunity, might also contribute to the observed antiviral effects [25]. While itraconazole also proved its antiviral potential in a 3D cell culture model for SARS-CoV-2 infection [23], an antiviral effect was not seen in the hamster infection model [61].

#### 2.4.2. Itraconazole Interferes with OSBP and OSBP-Related Proteins (ORP) Functionality

Azoles also impair cellular lipid metabolism via an inhibitory effect on oxysterol-binding protein 1 (OSBP) and on other proteins that belong to the OSBP-related proteins (ORP) family, and this property might add to their antiviral use. OSBP was first identified as an intracellular protein that binds cytosolic 25-hydroxycholesterol [62]. Beside its capacity to bind 25-hydroxycholesterol, OSBP orchestrates the formation of endoplasmic reticulum (ER)–Golgi complex membrane contact sites and thereby shuttles sterols into the Golgi and phosphatidylinositol-4-phosphate (PI4P) back to the ER [63]. OSBP and the family of OSBP-related proteins (ORP) share a lipid-binding domain that binds either a sterol or a nonsterol ligand as well as a PI4P-binding N-terminal pleckstrin homology (PH) domain [64,65]. Another binding motif found in many ORPs, including OSBP, is the FFAT-motif which interacts with the ER-resident VAMP-associated proteins (VAP) receptors [64,65]. Both motifs are involved in shaping the ER–Golgi or, in the case of some viruses, the ER–replication organelle (RO) contact sites and are considerably engaged in lipid transport through different organelles.

Enterovirus, dengue virus, and hepatitis C virus replication are reported to depend on ORP and OSBP [30,31]. Pharmacologic inhibition, siRNA knockdown, and rescue of replication by overexpression have demonstrated the importance of ORPs and OSBP for enterovirus replication and propagation [31]. The virus-induced accumulation of PI4P lipids drives the recruitment of OSBP to these contact sides, and the OSBP-mediated transport of cholesterol and PI4P is pivotal for the formation and functionality of the enterovirus RO [31,66,67,68]. In line, OSBP knockdown and treatment with 25-hydroxycholesterol, an inhibitor of the cholesterol-PI4P exchange, negatively affects virus replication [69]; however, the precise molecular mechanism remains unclear. Itraconazole directly binds OSBP [31], leading to increased PI4P levels at the Golgi (in uninfected cells) or the RO (in infected cells), while the accumulation of cholesterol at the RO is blocked [31].

#### 2.4.3. Targeting mTOR Signaling via Itraconazole

Itraconazole not only impairs lipid homeostasis, but also affects different signaling pathways, including mammalian target of rapamycin (mTOR), hedgehog, and Wnt signaling pathways that are hijacked by a broad range of viruses to drive the production of infectious particles.

The mammalian target of rapamycin (mTOR) signaling cascade is a pivotal signaling pathway that regulates apoptosis and counteracts stress-induced autophagy (such as, e.g., that elicited by viruses). Although different cellular locations for mTOR complex 1 (mTORC1) and mTORC2 have been reported, mTORC1 lysosomal localization appears critical for its ability to sense and respond to cell starvation [70]. Cholesterol was recently identified to promote the recruitment of mTORC1 to the lysosomal membrane [71] and the mTOR signaling cascade is regulated in a cholesterol-dependent manner [72].

Several viruses have evolved strategies to subvert the mTORC1 signaling network to drive their replication and propagation [73,74,75,76,77,78,79,80,81]. The Semliki Forest virus (SFV), Sindbis virus (SINV), and Chikungunya virus (CHIKV), members of the alphavirus family, cause different diseases but have in common that they encode nonstructural proteins (nsP) [82]. The activation of the PI3K/Akt/mTOR pathway is mediated through the phosphorylated and membrane-attached protein nsP3, which forms the viral replication complex upon virus internalization [83,84,85]. The activation of mTOR signaling is also fundamental for infection with the *Flaviviridae* West Nile virus (WNV), Japanese encephalitis virus (JEV) and dengue virus (DENV) [86,87,88]. *Flaviviridae* infection increases mTOR activity through a PI3K-dependent mechanism to maintain translation of its positive-sense RNA genome and also delays WNV-induced apoptosis [76,89,90]. The hepatitis C virus, another *Flaviviridae* member, increases phosphorylation of mTOR through the nonstructural protein 5A (NS5A) [91]. NS5A seems to activate PI3K/Akt signaling by directly binding PI3K [92,93]. The activation of the mTORC1 pathway by HCV has been linked to antiapoptotic signals that ensure cell survival and maintain persistence by promoting steady-state levels of virus replication [94,95]. Among the β-herpesvirus, the human cytomegalovirus (HCMV) maintains mTORC1 activation [96,97] through the expression of the two HCMV immediate early proteins, IEP72 and IEP86 [98]. Another persistent virus that tightly regulates mTOR signaling pathways is the human immunodeficiency virus type 1 (HIV-1). In dendritic cells, the HIV-1 envelope glycoprotein activates mTOR to prevent autophagy and to increase virus infection. Pharmacological treatment with rapamycin decreased viral spreading [99]. Another study implied that the HIV-1 protein Nef initiates mTOR activation which can be blocked by inhibitors of mTOR or PI3K [100,101], suggesting that drugs that modify the mTORC1 signaling pathway could act as anti-HIV-1 agents [102,103].

Well-balanced mTOR signaling is vitally important for IAV infection [104,105]. Thus, the pharmacological inhibition of the mTOR signaling axis might serve as potential antiviral target. In contrast to other azoles, itraconazole additionally inhibits mTOR signaling through affecting the upstream 5′-AMP-dependent protein kinase (AMPK) [106], which is activated upon an increased AMP/ATP ratio and serves as a regulator of cellular energy levels [107]. Once activated, AMPK inhibits mTOR signaling [108]. The activation of AMPK through itraconazole is a result of direct binding and inhibition of the mitochondrial Voltage-Dependent Anion Channel 1 (VDAC1), a critical regulator of mitochondrial metabolism, resulting in a drop in cellular energy levels [27]. Itraconazole treatment also impairs vascular endothelial growth factor receptor 2 (VEGFR2) functionality in endothelial cells, which is mostly due to altered VEGFR2 glycosylation, trafficking, and signaling [109]. As some viruses like human papillomaviruses or hepatitis viruses promote angiogenesis to facilitate optimal supply by nutrients [110,111,112], this might be an additional beneficial effect of itraconazole in antiviral strategy.

#### 2.4.4. Itraconazole, a Modulator of Hedgehog Signaling

Another signaling axis that is hijacked by viruses to promote their own replication and spreading is the hedgehog (Hh) signaling pathway. Some viruses, e.g., influenza viruses, interfere with the expression of hedgehog by directly modulating the specific activity of the transcriptional effector, glioma-associated oncogene homolog (GLI) [113]. The GLI family of zinc-finger transcription factors and Smoothened (Smo) are the signal transducers of the Sonic hedgehog (Shh) pathway [114]. Shh is secreted from cells and binds to the Patched 1 (Ptch1) receptor, which in the unbound state inhibits the activity of the transmembrane protein Smo [114]. Smo activation and GLI1 nuclear relocation drive the expression of genes involved in proliferation and apoptosis [114]. A recent study showed that the IAV nonstructural protein 1 (NS1) alters the expression of Hh target genes by directly modulating the specific activity of the transcriptional effector GLI [113]. Smelkinson et al. identified a point mutation (A122V) in the NS1 protein, which led to significantly accelerated lethality when incorporated into a mouse-adapted influenza-A virus [113].

Hh signaling is also associated with hepatitis B virus (HBV) and HCV infection. Patients suffering from chronic HBV and HCV infection display increased hepatocyte production of Hh ligands [115]. Hh pathway activation often occurs as a response to fibrogenic repair of liver damage due to chronic viral hepatitis [115,116]. The HBV protein HBx stimulates the GLI activation through protein stabilization and nuclear localization in liver cancer cells while the exact mechanism is not fully understood [117]. These data clearly showed the importance of Hh signaling for the outcome of viral infections.

Earlier studies proposed an inhibitory effect of itraconazole on Hh signaling by direct action on Smo [118,119]; however, recent research showed that itraconazole inhibits the expression of Shh and GLI1 proteins without affecting the expression of Ptch1 and Smo [120]. In the case of viral infection, itraconazole could subvert the ability of the virus to increase the host cell permissiveness for viral replication.

#### 2.4.5. Itraconazole and Its Inhibitory Effect on Wnt Signaling

The ancient and evolutionary-conserved Wnt signaling network contains two arms, the β-catenin-dependent, and the β-catenin-independent pathway [121]. While in the “off” state, β-catenin is degraded in the proteasome; the “on” state is initiated by binding of Wnt to a receptor called Frizzled (FZD) [122], leading to increased cytosolic β-catenin levels, and β-catenin translocation into the nucleus, where it orchestrates, together with other transcription factors, the expression of genes involved in differentiation and proliferation [121]. Depending on the receptor and ligand combinations, Wnt signaling can also activate signaling pathways independently of β-catenin, e.g., the calcium-dependent activation of PKC and Ca^2+^/calmodulin-dependent protein kinases II (Ca^2+^/CAMKII), resulting in changes in cell adhesion.

Viruses intervene with Wnt signaling by either epigenetic modification of Wnt gene expression or through interaction with specific Wnt pathway members, often resulting in the nuclear translocation of β-catenin and activation of Wnt signaling [122]. Both arms of Wnt signaling modulate the expression of genes that are required for the maintenance of viral pathogenesis such as for adenovirus and coxsackievirus B3 [122]. Furthermore, Wnt signaling is also involved in viruses-induced cancer development [123,124,125,126,127]. Given that diverse viruses affect this signaling pathway, Wnt signaling might be modulated in an antiviral manner. Indeed, itraconazole was found to negatively affect the expression of Wnt growth factor protein Wnt3A and to downregulate β-catenin, while increasing the levels of the endogenous Wnt inhibitor Axin-1 [128,129].

## 3. Conclusions

In most of the cases, the reported antiviral activities of the different antifungal agents are rather descriptive, thus it is not yet possible to formulate general principles of their antiviral activities (except for the azoles, which seem to act mostly via interference with the host cell cholesterol homeostasis, including the direct inhibitory binding to the endolysosomal cholesterol transporter NPC1). For most of the antifungals, the precise molecular targets, a prerequisite to an understanding of a drug’s mechanism(s) of action (MOA), remain to be uncovered. Moreover, an antifungal’s antiviral activity might most probably be associated with several host cell targets (see, for example, itraconazole), or might affect viral components (see, for example, micafungin). In this regard, the above-listed reports can only serve as starting points to accelerate the ultimate goal of developing tailored pharmaceuticals that recognize and interact with the respective molecular structure in the desired manner. However, the fact that a drug MOA is unknown or unclear does not mean that the drug holds no therapeutic potential. Rather, we would like to draw the readers’ attention to their antiviral modes of action (MoA), i.e., the observed lowered levels of viral replication, for the following reasons:

Repurposing clinically licensed drugs with well-known safety profiles (in this case, antifungals) that additionally target host cell factors required for virus entry, replication, or propagation might be a promising starting point for the development of novel prophylaxis and treatment approaches of viral infections. As they interfere with cellular metabolism and processes, such compounds are thought to cause fewer resistances, a key issue with direct antivirals. The combinatory use of such host-directed drugs with common antivirals could strengthen the antiviral effect and help to overcome viral infections. While drugs that directly target viral components are much more efficient to eliminate the pathogens, drugs that act on essential host cell factors are considered to circumvent the risk of resistance emergence. Combination therapy using two or more drugs to simultaneously hit multiple targets is, therefore, considered a key strategy to achieve therapeutic success at lower doses and a reduced likelihood of drug resistance development [130,131,132]. Targeting the virus/host interface via repurposing of azoles in combination with direct antivirals might thus provide a superior antiviral strategy [23,133]. Indeed, several studies have shown the benefit of combining azoles with antivirals [23,32,133]. In vitro studies revealed an additive effect of combinatory treatments with itraconazole or posaconazole and oseltamivir, a well-tolerated inhibitor of IAV neuraminidase. A synergistic antiviral effect was observed in SARS-CoV-2 infection models in vitro, when itraconazole was administered in combination with the antiviral remdesivir, a viral RNA-dependent RNA-polymerase inhibitor [23]. Similarly, a synergistic antiviral effect against HCMV was obtained with the combination of posaconazole and the anti-HCMV drug ganciclovir [32]. These data strongly argue for azoles as a useful element in combinatory treatments to combat certain viral infections. As already stated, itraconazole is a direct inhibitor of the endolysosomal cholesterol transporter NPC1 [47]. Because host cell cholesterol balance has been observed to exert a pivotal role in the infection cycles of several enveloped viruses including Ebola and influenza viruses [25,50,51], analyzing the relationships between the chemical structure of itraconazole and its biological activity (i.e., blocking NPC1 functionality) appears the path forward, and by understanding the structure–activity relationships (SAR), molecular docking simulation can then be used to rationally design novel antiviral derivatives.

Nevertheless, although beneficial effects were already observed as stated above, there are several issues to be considered:

**Bioavailability**—The low water solubility of the highly lipophilic azoles, including itraconazole, and the resulting poor bioavailability after oral application is a major disadvantage [134,135]. Itraconazole absorption critically depends on low pH, thus reduced gastric acidity caused by fasting conditions or medications (e.g., proton-pump inhibitors such as the widely used drug omeprazole) can considerably reduce the bioavailability and the absorption from the gastrointestinal tract [136], leading to high variability in the plasma levels of patients [134,137], and intake of acidic beverages is known to improve itraconazole absorption [138]. Indeed, a failure to reach the adequate serum concentrations intended in patients receiving systemic antifungal treatment has been observed [139].

**Safety**—Ideally, the therapeutically effective dose of a drug is much lower than the dose that leads to unwanted adverse effects. Nevertheless, several well-known and widely used drugs have a narrow therapeutic index and thus require frequent monitoring. Drug-induced organ injury is a considerable safety risk, with liver, heart, and kidney damage being the most common reasons for stopping the medication. Hepatotoxicity has been reported as a main adverse effect of antifungal treatment, more frequently in patients treated with azoles [140], and therapeutic drug monitoring has been recommended [141]. Again, SAR analyses might accelerate the development of engineered derivatives with enhanced bioavailability and safety profiles.

## Figures and Tables

**Table 1:** Overview of antifungal drugs, their mechanism of action, their clinical use, and a potential use as repurposed antivirals.

Antifungal Drug Family	Mechanism of Action	Clinical Use as Antifungals	Antiviral Potential
Polyenes	Bind sterol components and form pores, resulting in a compromised fungal plasma membrane [11].	Aspergillosis, cryptococcosis, candidiasis, zygomycosis, fusariosis, coccidioidomycosis, paracoccidioidomycosis, histoplasmosis, blastomycosis, mucormycosis, penicilliosis, and phaeohyphomycosis [11].	Japanese encephalitis virus [12], herpes simplex virus (HSV) [13], human immunodeficiency virus (HIV) [14], rubella virus [12], vesicular stomatitis virus (VSV) [15]
Flucytosine	Interferes with fungal nucleic acid synthesis [16].	Candidiasis, and cryptococcosis [16].	Not known
Echinocandins	Inhibit the fungal enzyme β1,3-glucan synthase, leading to incomplete fungal cell wall formation [17].	Aspergillosis, and candidiasis [17].	Chikungunya virus (CHIKV) [18], enteroviruses [19], dengue virus [20], SARS-CoV-2 [21], Sindbis virus (SINV) and Semliki Forest virus (SFV) [18]
Azoles	Primarily inhibit the fungal sterol biosynthesis, leading to compromised fungal membranes [22].	Aspergillosis, candidiasis, and cryptococcosis [22].	SARS-CoV-2 [23,24], influenza virus [25], Ebola virus [26,27,28], Parechovirus A3 [29], dengue virus [30], enteroviruses [31], human cytomegalovirus [32]

## Data Availability

Data sharing is not applicable to this article.
